# How endangered is sexual reproduction of high-mountain plants by summer frosts? Frost resistance, frequency of frost events and risk assessment

**DOI:** 10.1007/s00442-012-2581-8

**Published:** 2013-02-06

**Authors:** Ursula Ladinig, Jürgen Hacker, Gilbert Neuner, Johanna Wagner

**Affiliations:** Faculty of Biology, Institute of Botany, University of Innsbruck, Sternwartestraße 15, 6020 Innsbruck, Austria

**Keywords:** Alpine plants, Frost resistance, Ice nucleation, Reproductive development, Reproductive success

## Abstract

In temperate-zone mountains, summer frosts usually occur during unpredictable cold spells with snow-falls. Earlier studies have shown that vegetative aboveground organs of most high-mountain plants tolerate extracellular ice in the active state. However, little is known about the impact of frost on reproductive development and reproductive success. In common plant species from the European Alps (C*erastium uniflorum, Loiseleuria procumbens, Ranunculus glacialis, Rhododendron ferrugineum, Saxifraga bryoides, S. moschata, S. caesia*), differing in growth form, altitudinal distribution and phenology, frost resistance of reproductive and vegetative shoots was assessed in different reproductive stages. Intact plants were exposed to simulated night frosts between −2 and −14 °C in temperature-controlled freezers. Nucleation temperatures, freezing damage and subsequent reproductive success (fruit and seed set, seed germination) were determined. During all reproductive stages, reproductive shoots were significantly less frost resistant than vegetative shoots (mean difference for LT_50_ −4.2 ± 2.7 K). In most species, reproductive shoots were ice tolerant before bolting and during fruiting (mean LT_50_ −7 and −5.7 °C), but were ice sensitive during bolting and anthesis (mean LT_50_ around −4 °C). Only *R. glacialis* remained ice tolerant during all reproductive stages. Frost injury in reproductive shoots usually led to full fruit loss. Reproductive success of frost-treated but undamaged shoots did not differ significantly from control values. Assessing the frost damage risk on the basis of summer frost frequency and frost resistance shows that, in the alpine zone, low-statured species are rarely endangered as long as they are protected by snow. The situation is different in the subnival and nival zone, where frost-sensitive reproductive shoots may become frost damaged even when covered by snow. Unprotected individuals are at high risk of suffering from frost damage, particularly at higher elevations. It appears that ice tolerance in reproductive structures is an advantage but not an absolute precondition for colonizing high altitudes with frequent frost events.

## Introduction

High mountains are cold habitats where freezing temperatures occur throughout the year. Depending on the geographical location, moderate to strong frosts occur every night (mountains of the tropics and subtropics, arid mountains) or mainly during longer periods in winter (mountains of the temperate zone). Winter frost does not usually affect plants in mountains of the temperate zone as plants are mostly covered by snow where they experience more moderate and constant temperatures of between 0 and −5 °C (Cernusca 1976; Körner and Larcher 1988; Neuner et al. 1999; Larcher and Wagner 2009; Körner 2011). For larger plants protruding through the snow, winter frost hardening in most cases provides sufficient protection against frost damage (Sakai and Larcher 1987; Neuner 2007; Larcher et al. 2010). However, the situation becomes critical with the start of the growing season, when frost dehardened and actively growing tissues are exposed to unpredictable frost events (Taschler et al. 2004; Larcher et al. 2010; Neuner and Beikircher 2010). Episodic frosts during the growing season mostly occur during clear nights following snowfalls. Though small-statured mountain plants are usually sufficiently protected against frost damage when covered by snow, they may experience frost damage when the snow cover is shallow, absent at windblown ridges, or melts during the day (Körner 2003). During cold spells in summer (June–August), the free air temperature can cool to −5 °C at the timberline and to −15 °C in the nival zone in the European Alps (Larcher and Wagner 2009). Under these conditions, the somewhat higher dwarf shrubs and flowering stems that often overtop the vegetative parts of a plant are at a particularly high risk of becoming frost damaged (Larcher and Wagner 2004). Unpredictable summer cold spells in the main distribution zone of mountain plants have to be distinguished from regular frosts in the nival zone of the Alps where outposts of higher plants still occur. During the growing season, outside of cold snaps, plants experience freezing temperatures between 0 and −2 °C on about 70 % of days at 3,500 m a.s.l. and daily above 4,000 m a.s.l. (Larcher and Wagner 2009; Körner 2011). These conditions impose special requirements that are met by only a few specialists. Among these are, inter alia, the herbal species *Ranunculus glacialis* and the cushion plants *Androsace alpina*, *Saxifraga biflora*, *S. bryoides*, *S. moschata* and *S. oppositifolia,* the latter holding the altitudinal record in the European Alps at present (4,500 m a.s.l., Dom de Mischabel, Switzerland; Körner 2011).

Summer frost resistance of the vegetative aboveground organs of mountain plants has been well studied (e.g. Sakai and Otsuka 1970; Larcher and Wagner 1976; Squeo et al. 1991, 1996; Körner 2003; Taschler and Neuner 2004; Bannister et al. 2005; Bannister 2007; Larcher et al. 2010). At subzero temperatures, plants may either avoid freezing by supercooling or tolerate extracellular freezing and subsequent freeze-dehydration to a certain extent (Goldstein et al. 1985; Rada et al. 1987; Squeo et al. 1991; Hacker and Neuner 2008; Hacker et al. 2011; Neuner and Hacker 2012). As frost resistance is an adaptive trait, its extent differs according to the predictable environmental temperature regime (Sakai and Larcher 1987; Larcher 2005). In tropical mountains (e.g. Mt Kenia, Venezuelan Páramo), subtropical mountains (e.g. Andes of Northern Chile) and in arid mountains (e.g. Pamir), where strong night frosts regularly occur, most herbaceous and cushion plants survive at least −10 °C without frost damage (Tyurina 1957; Squeo et al. 1991, 1996; Beck 1994). In the humid-temperate mountains—where summer minima are not too extreme—plants are markedly less frost resistant. Bannister et al. (2005) and Bannister (2007) report the onset of damage at −6 to −7 °C for herbs and cushion plants from the New Zealand Alps. In the European Alps, initial damage occurs from about −4 °C in herbs and from about −6 °C in cushion plants (Larcher and Wagner 1976; Körner 2003; Taschler and Neuner 2004).

In contrast to the well-studied vegetative organs, practically no information is available about frost resistance of reproductive structures in mountain plants. Knowledge in this field is particularly important, as flowering and seed formation are essential functions which ensure population turnover and determine the distribution potential of a species. As shown for lowland plants, reproductive stages are the most vulnerable phases in the annual cycle of a plant (for review, see Larcher 1985; Sakai and Larcher 1987). Thus, it can be expected that actively growing reproductive structures of mountain plants are at greater risk of frost damage than are vegetative structures. Seed loss is regularly observed following spring and summer frosts in temperate zone mountains (Inouye et al. 2002; Inouye 2008; Ladinig and Wagner 2007), and in Scandinavian mountains (Molau 1996).

The aim of this study was to compare summer frost resistance of reproductive shoots to aboveground vegetative foliated shoots (for definition of the term “shoot”, see “Materials and methods”), to reveal the impact of frost on reproductive success and to carry out a risk assessment for seven common plant species from the European Central Alps. Species which differed in their growth form (dwarf shrubs, herbs, cushion plants), phenology (early, mid-, late flowering), and altitudinal distribution range (subalpine, alpine, subnival, nival; zonation according to Ozenda 1988) were selected. Our study extends on an earlier in situ study investigating summer frost resistance in leaves of major alpine plant growth forms in the European Alps in relation to their upper distribution boundary (Taschler and Neuner 2004).

We wanted to know in detail to what extent unpredictable, short frost events impair reproductive shoots at different developmental stages (bud stage, bolting, anthesis, early and late fruiting stage) and what consequences these events might have with regard to reproductive success (fruit/flower ratio, seed/ovule ratio, seed germination). To this end, intact potted plants were exposed to simulated night frosts of different intensities in temperature-controlled freezing chambers. During simulated freezing, the rooting space was thermally insulated allowing frost to act only on the aboveground organs as is the case during short frost episodes at the natural sites. To obtain information regarding whether reproductive shoots are ice tolerant (i.e. they tolerate extracellular freezing and freeze-dehydration) or ice susceptible (any tissue freezing is lethal), ice nucleation temperatures were recorded. Following the frost treatments at different reproductive stages, the extent of frost damage in reproductive and vegetative shoots was analysed, and the effect of frost on reproductive success was determined. By linking data for frost resistance with site temperatures, the potential risk of suffering frost damage from episodic frosts during different reproductive stages was assessed.

We addressed the following questions: (1) are there differences in frost resistance between vegetative and reproductive shoots within a species; (2) are there differences in frost susceptibility among the main reproductive stages (bud stage, flowering, fruiting); (3) is there a relationship between altitudinal distribution, growth form and phenology (early, mid- and late flowering) on the one hand and frost resistance on the other; and (4) at what frequency and intensity do frost events occur in the different habitats and what is the probability the species will suffer from frost damage during different reproductive stages?

We expected that, similar to what has been shown for lowland plants (Sakai and Larcher 1987), reproductive shoots of mountain plants would be at greater risk of suffering frost damage than vegetative aboveground shoots, and that reproductive shoots around anthesis would be particularly frost susceptible. We further assumed that nival plant species would tolerate frosts better than species restricted to the alpine zone, and that early flowering species would be less frost-susceptible than later flowering species.

## Materials and methods

### Plant material and collection sites

The seven study species and their characteristics are summarized in Table 1. All species occur commonly in and are typical of their respective habitat. *Rhododendron ferrugineum* L., a mid-season flowering shrub up to 90 cm in height, often dominates the dwarf shrub heath at the timberline ecotone and in the lower alpine zone of the Central European Alps (Ellenberg and Leuschner 2010). *Loiseleuria procumbens* L., a prostrate-growing, early-flowering dwarf shrub, is representative of *Loiseleuria* associations in the subalpine and alpine zone of the Central European Alps (Ellenberg and Leuschner 2010). The species often grows on windblown ridges with little snow protection. *Cerastium uniflorum* Clairv., *Ranunculus glacialis* L. and *Saxifraga bryoides* L., are typical of the plant assemblages from the subnival (i.e. the alpine-nival ecotone; Pauli et al. 1999) to the nival zone throughout the Central European Alps (Gottfried et al. 2011). *C. uniflorum* is a hemicryptophyte, forming loose cushions through annual shoot growth; it is mid- to late flowering. *R. glacialis* is an arctic-alpine herbaceous rhizome plant with particularly fast reproductive development (Wagner et al. 2010, 2012). *S. bryoides* forms firm cushions with densely arranged short-stem shoots and is mid- to late flowering (Ladinig and Wagner 2007, 2009). The cushion plant *Saxifraga caesia* L. is widely distributed in the alpine zone of limestone mountains within the association Caricetum firmae (Kaplan 1995); *Saxifraga moschata* Wulfen is abundant from the alpine to the nival zone on base-rich substrates and shows a high phenological plasticity (Ladinig and Wagner 2005).

**Table 1:** Characteristics of the investigated species

Growth form	Plant species	Abbreviation	Mountain belt^a^	Vertical distribution (m a.s.l.) in the Alps^b^	Sampling site^c^	Life form^d^	Type of reproductive shoot	Flowering time
Dwarf shrubs	*Loiseleuria procumbens* L.	*Loi pro*	Alpine	1,800–2,800	P	Chamaephyte	Inflorescence	Early (May–June)
	*Rhododendron ferrugineum* L.	*Rho ferr*	Subalpine-alpine	700–2,500 (3,000)	P	Phanerophyte	Inflorescence	Mid (June–July)
Perennial herbs	*Cerastium uniflorum* Clairv	*Cer uni*	Subnival–nival	2,000–3,500	S	Hemicryptophyte	Terminal flower	Late (July–August)
	*Ranunculus glacialis* L.	*Ran gla*	Subnival–nival	>2,000 (4,275)	S	Cryptophyte	Inflorescence	Early (June–July)
Cushion plants	*Saxifraga bryoides* L.	*Sax bry*	Subnival–nival	>2,000 m (4,200)	S	Chamaephyte	Solitary flower	Late (July–August)
	*Saxifraga caesia* L.	*Sax cae*	Alpine	1,600–2,600 (3,000)	H	Chamaephyte	Inflorescence	Late (July–August)
	*Saxifraga moschata* Wulfen	*Sax mos*	Alpine–nival	>1,600 (4,200)	H	Chamaephyte	Inflorescence	Mid (June–July)

^a^Mountain belt in the European alps: *subnival* alpine-nival ecotone (Pauli et al. 1999), *nival* ice-free areas within the glacier zone

^b^Vertical distribution according to Hegi (1975), Kaplan (1995), Landolt (1992) and Zimmermann (1975); numbers in parentheses give the highest localities in the Swiss Alps

^c^Sampling site *H* Mt Hafelekar (2,350 m a.s.l.), *P* Mt Patscherkofel (1,950 m a.s.l.), *S* Stubai Glacier foreland (2,880 m a.s.l.)

^d^According to Raunkiaer (1934)

Frost treatments on cushion plants and herbs were carried out on intact potted plants or, in the case of dwarf shrubs, directly in the field using the method of Taschler and Neuner (2004). Individual plants of *R. glacialis*, *S*. *bryoides* and *C. uniflorum* originated from a subnival site in the foreland of the Stubai Glacier (2,880 m a.s.l., 46° 59′N, 11° 07′E) in the Tyrolean Central Alps. *S. moschata* and *S. caesia* were collected at an alpine site in the calcareous mountain range north of Innsbruck (Mt Hafelekar, 2,350 m a.s.l., 47°18′N, 11°23′E). For each reproductive stage, whole plants were excavated with root bales and potted into plastic pots (8 × 8 cm) in original soil (alpine site: alpine pitch Rendsina, Rehder 1976; subnival site: siliceous scree, Huber et al. 2007). Plants were transported in cooling boxes to the laboratory within 1 h (alpine sites), and 2 h (subnival sites), respectively.

To check for the actual state of frost resistance, plants were frost-treated either immediately on return to the laboratory or after storage in a growth chamber (photoperiod 16/8 h, temperature 10/5 °C; PGC-GL; Percival Scientific, Perry, IA, USA) for no more than 3 days. Control plants were kept in the growth chamber throughout. The woody shrubs of *R. ferrugineum* and *L. procumbens* could not be excavated and therefore were frost-treated in situ at the timberline (1,950 m a.s.l) on Mt Patscherkofel (47°12′N, 11°27′E; for details, see Larcher et al. 1975) about 6 km south of Innsbruck.

### Reproductive stages

During the 2008 and 2009 growing seasons, frost resistance of aboveground vegetative and reproductive shoots was determined in the following reproductive stages: bud stage *b1* (reproductive buds before bolting), bud stage *b2* (inflorescences during bolting, flower buds still closed), anthesis *a*, fruit stage *f1* (infructescences during early fruit development, seeds undergo histogenesis) and fruit stage *f2* (infructescences during late fruit development, seeds in the maturation phase). In *C. uniflorum, L. procumbens* and *R.* *glacialis* only bud stage *b2* was investigated. Depending on the species and its morphology, frost resistance data for *vegetative shoots* refer to the following vegetative aboveground organs: mature stems and leaves of the evergreen shrubs *R. ferrugineum* and *L. procumbens*, newly forming stems and leaves of the hemicryptophyte *C. uniflorum*, leaves of the cryptophyte *R. glacialis*, and leafy short-stem shoots of the *Saxifraga* cushions. Depending on the state of reproductive development, the term *reproductive shoot* stands for the inflorescence bud, the inflorescence and the infructescence including the peduncle (*R.* *glacialis*, *S. caesia*, *S. moschata*, *R. ferrugineum*, *L. procumbens*), or for single flower buds, flowers and fruits including the stalks (*C. uniflorum,* *S. bryoides*).

### Simulating night frosts

Freezing treatments were conducted in temperature-controlled chest freezers (GT2102; Liebherr, Lienz, Austria). The freezers are modified in order to expose plant samples to controlled temperature runs. The adjustment of the actual target temperature is achieved by electric bulbs that are turned on and off and act as heat sources. The lamps are in a separate compartment of the chest freezer, so plants are frost-treated in complete darkness. Ventilators provide air circulation and prevent thermal gradients. The control unit of the system is a PC including measurement modules for temperature measurements with thermocouples (National Instruments, USA). The freezing system is directed by a cooling software program written by J. Hacker in LabView (National Instruments). The program allows independent temperature runs to be compiled in each of the chest freezers. During the temperature treatments, only the aboveground parts of the potted plants were exposed to the freezing temperatures. Roots were kept at more moderate temperatures of between −2 °C and +3.8 °C by means of a thermally insulated and heated root chamber (12 W heating power, realized by a power resistor and ventilation). In situ frost treatments of the dwarf shrubs *R. ferrugineum* and *L. procumbens* were conducted in portable ventilated and thermally insulated freezing chambers (for detailed description, see Taschler and Neuner 2004 and http://www.othmar-buchner.at/pages/p2.htm).

For each plant species and reproductive stage, target freezing temperatures were set in 2-K steps (−2, −4, −6, −8, −10, −12, and −14 °C) across the full temperature range from 0 to 100 % frost damage. Test series were repeated 3–6 times with 2–6 individuals per temperature step and 2–5 control individuals per test series. In total, 6–15 individuals were treated per temperature step. In a single freezing experiment, cooling occurred at a rate of 2 K h^−1^ down to the target temperature, which roughly corresponds to the maximum leaf cooling rates in the subzero temperature range during natural freezing events in alpine summer (Larcher et al. 2010; Neuner and Hacker 2012). Plants remained at the target temperature for 4 h and were thereafter thawed again at a rate of 2 K h^−1^. Plant temperatures were continuously recorded using fine-wire copper-constantan thermocouples (welding spot diameter 0.15 mm). The welding spots were closely attached to leaves and stems with a special adhesive tape (Transpore; 3 M international) or inserted into flower buds and open flowers without damaging the structures. For each freezing series, 32 thermocouples were mounted. Ice nucleation temperatures (NT) were determined graphically as the lowest temperature immediately before the onset of the freezing exotherm. Temperatures are presented in °C and temperature differences in Kelvin (K) as is the custom in bioclimatology (Leuzinger et al. 2010).

### Assessment of frost damage

After the frost treatments, plants were kept in a growth chamber for 1–4 days (photoperiod 16/8 h, temperature 15/5 °C, PGC-GL; Percival Scientific), and then—together with the control plants—returned to the natural growing sites. There, pots were embedded into the soil and regularly watered. About 2 weeks after a frost treatment, frost damage was assessed on each individual. For vegetative shoots, the percentage of visually damaged areas was assessed. Reproductive buds, inflorescences and infructescences of *R. glacialis* and the saxifrages were either killed or remained undamaged and developed further. Thus, the extent of damage was expressed as the percentage of dead whole reproductive shoots per individual. In the reproductive shoots of *L. procumbens* and *R. ferrugineum*, partial frost damage also occurred, i.e. single flower buds, flowers and young fruits within an inflorescence/infructescence remained undamaged and developed further; the extent of damage was therefore expressed as the percentage of dead flowers/fruits per inflorescence/infructescence. Frost damage to senescing and partly lignified inflorescences in the *f2* stage was not unambiguously discernable and therefore was not evaluated. Equally, frost damage in the mostly tiny seeds could not be assessed by visual scoring. In *C. uniflorum* and *S. caesia*, viability of seeds was assessed via germinability (see next paragraph “Reproductive success”). Germination tests of the remaining species were not evaluable because of complex dormancy mechanisms (Schwienbacher et al. 2011), which led to very low levels of germination even in control seeds.

For each test series, the percentage damage in vegetative and reproductive shoots of each individual was separately plotted against the treatment temperature (i.e. the mean temperature recorded by the thermocouples), and a logistic function was fitted (Boltzmann function) using the software OriginPro 7 g (OriginLab, Northampton, MA, USA). The following threshold values for frost damage were determined: LT_10_, LT_50_, LT_90_ (temperatures at 10, 50 and 90 % frost damage) and LT_100_ (highest temperature causing 100 % frost damage in the tested individuals). LT_10_, LT_50_ and LT_90_ were calculated as parameters from the logistic function and read from the curve-fitting protocol (cf. Taschler et al. 2004).

### Reproductive success

Reproductive success was evaluated by determining the fruit/flower ratio (Fr/Fl ratio), the seed/ovule ratio (S/O ratio), and seed germinability, in both frost-treated and control plants. For the Fr/Fl ratio, the fraction of flower buds and flowers that matured fruits was assessed for each individual. In the multi-flowered inflorescences of *S. caesia* and *S. moschata*, only the terminal flowers were considered because number and success of lateral flowers vary considerably and therefore cannot be reliably evaluated. To determine the S/O ratio, all intact appearing fruits that had developed from flower buds, flowers and early fruits in control and in frost-treated individuals were harvested shortly before capsule dehiscence and fixed in Carnoy solution (96 % ethanol, glacial acetic acid, 3:1). Apparently intact seeds (S) and undeveloped ovules or seeds (together referred to as O) per fruit capsule (in *R. glacialis* per aggregate fruit) were counted under a stereomicroscope, and the S/O ratio was calculated. Depending on the plant species, 11–181 fruits from 9–58 individuals were examined per developmental stage. For control plants, 30–100 fruits from 7–21 individuals were examined.

To test frost effects on maturing seeds of *C. uniflorum* and *S. caesia* (stage *f2*), frost-treated plants together with control plants were kept in growth chambers (photoperiod 16/8 h, temperature 15/5 °C, PGC-GL; Percival Scientific) until full fruit maturity. This state was usually reached within 10 days. Seeds were cold-stratified both in the laboratory and at the natural sites prior to the germination tests. For laboratory tests, 170–550 mature seeds per test temperature (2-K steps between −2 and −12/−14 °C) from 3 to 20 fruits from 2 to 4 individuals were placed in Petri dishes on moist filter paper and stored in a cooling chamber at 4 °C in the dark for 6 months. Permanent humidity inside the dishes was ensured by placing the filter paper on glass beads immersed in water. After cold stratification, dishes were transferred into a growth chamber (photoperiod 16/8 h, temperature 20/5 °C, PGC-GL; Percival Scientific). Seed germination (radicle at least as long as the seed or longer) was recorded at regular intervals over a period of 6 months. In “Results”, the final level of germination is given.

For in situ germination tests, 50–600 mature seeds per species and temperature step were enclosed in gauze bags (50–100 seeds per bag), and temporarily stored dry at 5 °C until placing them at the natural sites below shallow soil in October 2009. Seeds passed the winter under the snow and were checked for germination about 2 weeks after snow melt in 2010 and then after a further 4–5 months at the respective mountain sites in the laboratory under a stereomicroscope.

### Site temperatures

Microenvironmental temperature data from the alpine site (2,350 m a.s.l., Mt Hafelekar), the subnival site (2,880 m a.s.l., Stubai Glacier) and a nival site (3,450 m a.s.l., Mt Brunnenkogel, 46°55′N, 10°52′E), where the investigated plant species respectively occur, have been recorded from 2002 to 2010 at hourly intervals throughout the years using small data loggers (Tidbit; Onset, Bourne, MA, USA). At each site, three temperature loggers were placed near the ground in plant cushions or below the leaves of *R. glacialis*, and, during the growing season, shaded by white plastic caps to avoid overheating. Air temperatures (2 m) from standard weather stations at the same elevation in the Tyrolean Alps were provided by the Central Institute for Meteorology and Geodynamics, Austria (ZAMG), for Mt Patscherkofel (2,246 m a.s.l., 47°12′31″N, 11°27′38″E), Pitztal Glacier (2,840 m a.s.l., 46°55′36″N, 10°52′46″E), and Mt Brunnenkogel (3,450 m a.s.l., 46°54′45″N, 10°51′40″E).

From the timberline site (1,950 m a.s.l.), we have temperature data from 1998 to 2004 from an automatic weather station (CR10; Campbell Scientific, Logan, UT, USA) in the Alpine Garden of the Institute of Botany, University Innsbruck (operated by G. Neuner). Canopy temperatures of *R. ferrugineum* shrubs at 50–70 cm height and 2-m air temperatures in immediate proximity were measured at 30-s intervals using thermocouples.

For each site, the absolute daily minimum temperature during the multiannual measurement period was determined in the plant canopy near the ground and 2 m above the ground for the growing period (May–August). Additionally, the frequency of freezing temperatures in the ranges of −1 to −1.99, −2 to −2.99, −3 to −4.99, −5 to −6.99 and ≤−7 °C was calculated for the first, middle and last third of each month (cf. Fig. 5). Based on the frequency of the different classes of frost temperatures, the empirical probability (*EP*, range 0–1) that reproductive shoots exceed the threshold value LT_10_ for frost damage was determined for each species and reproductive stage (cf. Table 5).

In addition to the long-term temperature records, diurnal changes of plant temperatures near the ground, and at a height of 5 cm, and 2-m air temperatures were recorded with fine-wire thermocouples in 2009 at the subnival site (2,880 m a.s.l., Stubai Glacier). Thermocouples were connected to data loggers (CR10; Campell Scientific) that collected temperature records from the sensors every 5 min.

### Statistics

Statistical differences in frost resistance (LT_50_) among species, species groups, reproductive stages, and between vegetative and reproductive shoots, were tested either by one-way ANOVA followed by the Bonferoni post hoc test or by *t* test. To study relationships between treatment temperature and S/O ratio, and between treatment temperature and seed germinability, correlation analyses (Pearson) were carried out. In all tests, the critical level of significance was *α* = 0.05. All analyses were carried out using the statistical package IBM SPSS Statistics 18 (IBM, New York, USA).

## Results

### Frost resistance of vegetative shoots

In most species, frost resistance of vegetative shoots did not significantly change during the growing season. Therefore, a mean summer frost resistance was calculated from all data obtained at different stages of reproductive development (Table 2). Exceptions were the woody plants *L. procumbens* and *R. ferrugineum*, whose expanding young shoots are particularly frost susceptible. In *S. moschata*, frost resistance of short-stem shoots significantly decreased in the transition from stage *b1* to *b2*, but remained approximately the same during the following reproductive stages.

**Table 2:** Frost resistance of vegetative shoots of the investigated plant species

Growth form	Species		Reproductive state	LT_10_ (°C)	LT_50_ (°C)	LT_90_ (°C)	LT_10_–LT_90_ (K)	LT_100 total_ (°C)
Dwarf shrubs	*Loi pro*	*veg e*	*b2, a, f1*	−5.7 ± 0.3	−5.9 ± 6.2	−6.2 ± 0.4	0.5 ± 0.1	−9.0
		*veg m*	*b2, a, f1*	−7.3 ± 2.0^ab^	−9.2 ± 2.0^ab^	−10.7 ± 2.9^a^	2.9 ± 2.0^ab^	−12.8
	*Rho ferr*	*veg e*	*b2, a, f1*	−3.4^A^	−3.6^A^	ND	ND	−4.1^A^
		*veg m*	*b1, b2, a, f1*	−5.9 ± 1.4^a^	−8.4 ± 1.4^a^	−10.3 ± 1.8^a^	3.9 ± 1.7^ab^	−11.5
Herbs	*Cer uni*		*b2, a, f1*	−6.9 ± 1.9^a^	−9.2 ± 1.5^ab^	−11.4 ± 1.4^a^	4.5 ± 1.5^ab^	−15.3
	*Ran gla*		*b2, a, f1*	−6.8 ± 1.4^a^	−9.2 ± 1.3^ab^	−11.7 ± 2.4^a^	4.9 ± 3.0^a^	−14.1
Cushion plants	*Sax bry*		*b1, b2, a, f1*	−10.6 ± 2.0^c^	−12.3 ± 0.5^c^	−13.9 ± 1.7^c^	3.3 ± 2.3^ab^	ND
	*Sax cae*		*b1, b2, a, f1*	−9.1 ± 0.9^bc^	−10.1 ± 0.7^b^	−11.6 ± 2.3^a^	2.4 ± 1.4^bc^	−13.0
	*Sax mos*		*b1*	−10.7 ± 0.9^c^	−12.0 ± 0.1^c^	−13.4 ± 0.8^c^	2.7 ± 1.6^b^	−16.0
			*b2, a, f1*	−6.0 ± 1.7^a^	−8.1 ± 1.1^ab^	−10.1 ± 1.0^a^	4.1 ± 1.4^ab^	−13.9

Values show the mean summer frost resistance (LT_10_, LT_50_, LT_90_, LT_100_), and the mean temperature range between LT_10_ and LT_90_ for the reproductive phases (*b1* before bolting; *b2* bolting; *a* anthesis; *f1* early fruit development). For the woody plants *L. procumbens* and *R. ferrugineum*, mean summer frost resistance only refers to mature vegetative shoots (*veg m*), frost resistance of expanding young shoots (*veg e*) is listed separately. In *S. moschata*, vegetative short-stem shoots are significantly frost hardier during *b1* (*p* < 0.001) than during later reproductive stages

Different lower case letters in each column indicate significant differences in frost resistance of vegetative shoots among species within the threshold values for frost damage LT_10_, LT_50_, LT_90_ and LT_10_–LT_90_ (one-way ANOVA, Bonferroni post hoc comparison, *α* = 0.05)

^A^Data from Taschler et al. 2004

*ND* not determined

**Fig. 1:**
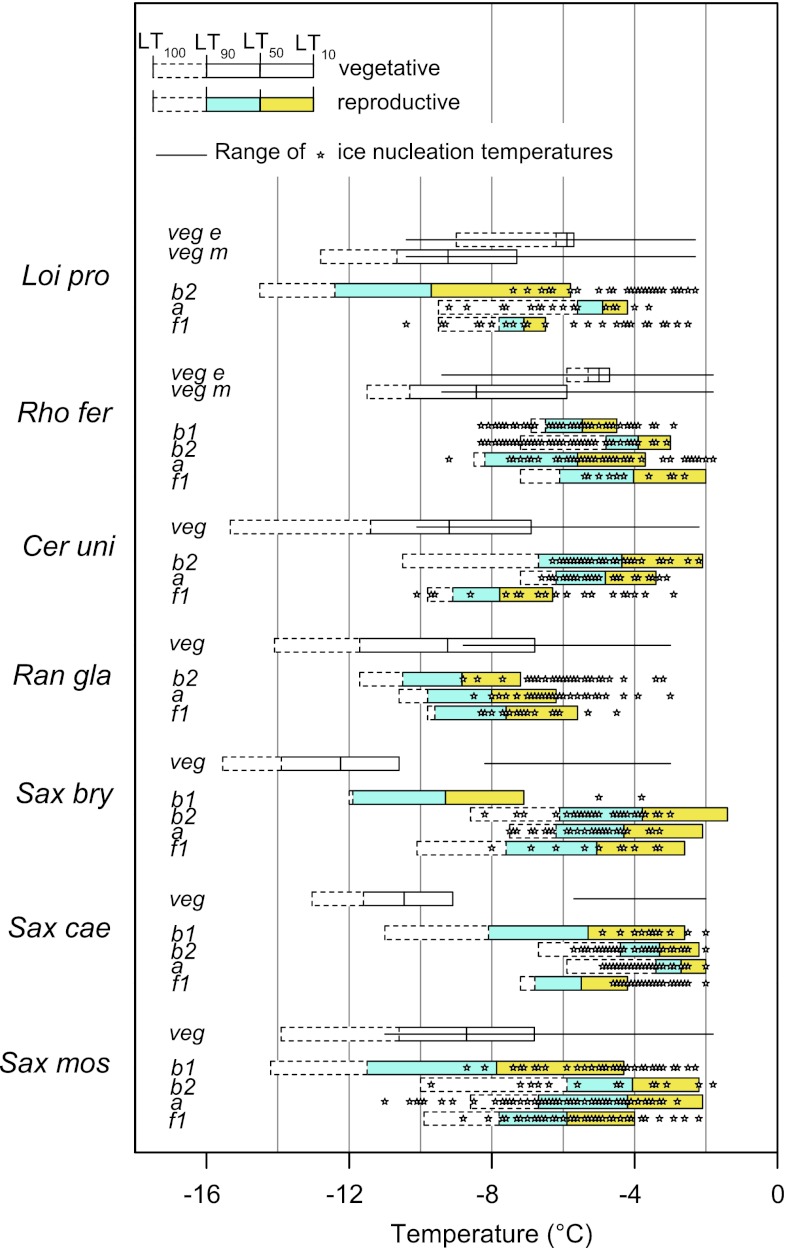
Mean summer frost resistance of reproductive shoots in different reproductive stages (*b1* before bolting; *b2* bolting; *a* anthesis; *f1* early fruit development), and vegetative shoots (*veg*). For *Loi pro* and *Rho fer* vegetative expanding (*veg e*) and vegetative mature shoots (*veg m*) are indicated separately. *Horizontal bars* range from LT_10_ to LT_100_ (from *right* to *left*). *Vertical lines inside bars* mark LT_50_ and LT_90_. For reproductive shoots, the range between LT_10_ and LT_50_ is displayed in *yellow*, the range between LT_50_ and LT_90_ in *blue*. Ice nucleation temperatures are indicated as single events by *asterisks* (reproductive shoots) or as range (vegetative shoots, *line within the bars*). For the abbreviations of species names see Table 1. In all investigated species, vegetative shoots were significantly frost hardier than reproductive shoots (comparison between LT_50_ values across all developmental stages; *Loi pro*: *p* = 0.004; *Rho fer*: *p* < 0.001; *Cer uni*: *p* < 0.001; *Sax bry*: *p* < 0.001; *Sax cae*: *p* < 0.001; *Sax mos*: *p* < 0.001; *t* test) except for *Ran gla* (n.s.). For statistical differences among reproductive stages, see Table 3

**Fig. 2:**
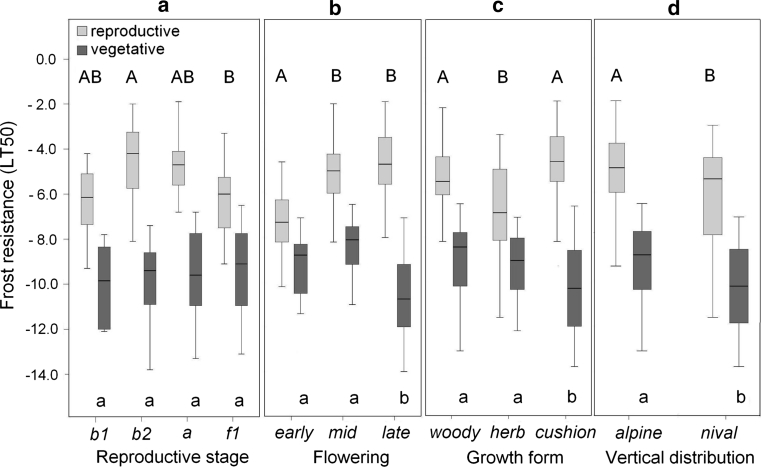
Summer frost resistance (LT_50_) of vegetative shoots (*dark grey bars*) and of reproductive shoots (*light grey bars*). Data for the species were pooled and grouped by **a** different reproductive stages **b** different flowering times, **c** different growth forms, and **d** different habitats. *Saxifraga moschata* which occurs from the alpine to the nival zone was assigned to the alpine group, as the investigated individuals originate from an alpine site. *Box plots* show the median (*line inside the box*), the 25th and 75th percentile (*extent of box*), and the whiskers range from maximum to minimum value. *Different letters* within subfigures indicate statistical differences among different groups for vegetative shoots (*lower case letters*) and for reproductive shoots (*capital letters*); (one-way ANOVA:** a**–**c**; *t* test **d**). Mean values of LT_50_ are significantly different between reproductive and vegetative shoots in all groups (*p* ≤ 0.001, *t* test)

Summer frost resistance among species differed significantly (Table 2; Fig. 1) and in the case of LT_50_ ranged from −8.1 °C (*S. moschata*) to −12.3 °C (*S. bryoides*). When all species were taken together, summer frost resistance of vegetative shoots (without expanding new shoots of the woody species) was not significantly influenced by the reproductive stage (Fig. 2a) but was higher in late flowering species than in early and mid flowering species (Fig. 2b; *p* ≤ 0.009, one-way ANOVA). Cushion plants tolerated lower temperatures than herbs and woody species (Fig. 2c; *p* ≤ 0.012, one-way ANOVA). With respect to the altitudinal distribution range, nival species were frost hardier than species of the alpine zone (Fig. 2d; *p* = 0.001, *t* test). The temperature span over which frost damage developed (LT_10–90_) was 2.4–4.9 K and did not differ significantly among most species (Table 2). In all species at all reproductive stages, extracellular ice formation was tolerated by the vegetative organs, as shown by the range of nucleation temperatures which mostly began at higher temperatures than first frost damage (Fig. 1).

### Frost resistance of reproductive shoots at different stages of development

The capacity of reproductive shoots to survive frost during the growing season strongly depended on the stage of development (Fig. 1; Table 3). Inflorescence/flower buds of cushion plants before bolting (*b1*) turned out to be the most frost resistant reproductive stage with LT_50_ values between −5.3 °C (*S. caesia)* and −9.3 °C (*S. bryoides)*. First frost damage (LT_10_) became visible at freezing temperatures 2–3 K lower than the highest ice nucleation temperature, indicating that *b1* buds are ice-tolerant.

**Table 3:** Summer frost resistance (LT ± SD, °C), temperature span between LT_10_ and LT_90_, and the range of ice nucleation temperature NT min/max and Δ min–max (in parentheses) in reproductive shoots of the investigated plant species at different stages of reproductive development (*b1* before bolting; *b2* bolting; *a* anthesis; *f1* early fruit development)

Stage	Frost resistance	Dwarf shrubs		Herbs		Cushion plants		
		*Loi pro*	*Rho ferr*	*Cer uni*	*Ran gla*	*Sax bry*	*Sax cae*	*Sax mos*
*b1*	**LT**_**50**_	ND	**−5.5** **±** **0.9 a,A**	ND	ND	**−9.3** **±** **0.8 a,A**	**−5.3** **±** **1.6 a,A**	**−7.9** **±** **1.6 a,A**
	LT_10_	ND	−4.5 ± 1.0	ND	ND	−7.1	−2.6 ± 1.3	−4.3 ± 0.7
	LT_90_	ND	−6.5 ± 1.2	ND	ND	−11.9	−8.1 ± 1.9	−11.5 ± 3.6
	LT_100_	ND	−7.6	ND	ND	−12	−11.0	−14.2
	Δ LT_10_–LT_90_	ND	2.3 ± 3.8	ND	ND	4.8	5.6 ± 0.6	7.2 ± 4.0
	*NT min/max* (Δ)	ND	−*2.9/*−*8.3* (*5.4*)	ND	ND	−*3.8/*−*5.0* (*1.2*)	−*2.0/*−*4.9* (*2.9*)	−*2.3/*−*8.7* (*6.4*)
*b2*	**LT**_**50**_	**−9.7** **±** **1.2 b,c,B**	**−3.9** **±** **1.7 a,A**	**−4.4** **±** **0.4 a,A**	**−8.8** **±** **1.9 b,A**	**−3.8** **±** **0.8 a,B**	**−3.3** **±** **0.8 a,B**	**−4.1** **±** **1.9 a,B**
	LT_10_	−5.8	−3.0 ± 1.3	−2.1 ± 0.7	−7.2 ± 1.7	−1.4 ± 0.8	−2.2 ± 0.7	−2.2 ± 1.1
	LT_90_	−12.4 ± 1.2	−4.8 ± 2.7	−6.7 ± 1.9	−10.5 ± 1.9	−6.1 ± 1.8	−4.4 ± 0.8	−5.9 ± 2.9
	LT_100_	−14.5	−7.2	−10.5	−11.7	−8.6	−6.7	−10.0
	Δ LT_10_–LT_90_	6.6	1.7 ± 4.4	4.6 ± 1.4	3.2 ± 0.9	4.7 ± 2.2	2.1 ± 1.6	3.7 ± 2.5
	*NT min/max* (Δ)	−*3.6/*−*7.4* (*3.8*)	−*3.1/*−*8.3* (*5.2*)	−*2.2/*−*6.3* (*4.1*)	−*3.0/*−*8.8* (*5.8*)	−*3.0/*−*8.2* (*5.2*)	−*2.0/*−*5.7* (*2.7*)	−*1.8/*−*9.7* (*7.9*)
*a*	**LT**_**50**_	**−4.9** **±** **0.3 a b,A**	**−5.6** **±** **0.2 b,A**	**−4.8** **±** **0.4 ab,A**	**−8.0** **±** **1.2 c,A**	**−4.3** **±** **0.6 a,B**	**−2.7** **±** **0.9 a,B**	**−4,2** **±** **0.5 a b,B**
	LT_10_	−4.2 ± 0.5	−3.7 ± 1.0	−3.4 ± 0.9	−6.2 ± 1.9	−2.1 ± 0.5	−2.0 ± 0.9	−2.1 ± 1.0
	LT_90_	−5.6 ± 0.3	−8.2 ± 1.3	−6.2 ± 1.3	−9.8 ± 2.3	−6.2 ± 1.1	−3.4 ± 1.0	−6.7 ± 1.3
	LT_100_	−9.5	−8.5	−7.2	−10.6	−7.2	−5.9	−8.6
	Δ LT_10_–LT_90_	1.4 ± 0.6	4.5 ± 2.3	2.8 ± 2.6	3.6 ± 3.7	3.8 ± 1.2	1.4 ± 2.7	4.9 ± 2.2
	*NT min/max* (Δ)	−*3.6/*−*9.2 (5.6*)	−*1.8/*−*9.2* (*7.4*)	−*3.1/*−*6.6* (*3.5*)	−*3.0/*−*8.5* (*5.5*)	−*3.3/*−*7.5* (*4.2*)	−*2.0/*−*4.9* (*2.9*)	−*2.8/*−*11.0* (*8.2*)
*f1*	**LT**_**50**_	**−7.1** **±** **0.9 a,B**	**−4.0** **±** **0.7 a,A**	**−7.8** **±** **1.1 a,B**	**−7.6** **±** **0.4 a,A**	**−5.1** **±** **0.8 a,A**	**−5.5** **±** **0.5 a,A**	**−5.9** **±** **0.9 a,A**
	LT_10_	−6.5 ± 2.0	−2.0 ± 0.3	−6.3 ± 1.4	−5.6 ± 1.3	−2.6 ± 1.9	−4.2 ± 1.0	−4.0 ± 1.6
	LT_90_	−7.8 ± 1.0	−6.1 ± 1.1	−9.1 ± 1.1	−9.6 ± 1.4	−7.5 ± 1.4	−6.8 ± 0.3	−7.8 ± 1.1
	LT_100_	−9.5	−7.2	−9.8	−9.8	−10.1	−7.2	−9.9
	Δ LT_10_–LT_90_	1.4 ± 2.2	4.2 ± 0.9	2.8 ± 1.4	3.9 ± 1.0	4.8 ± 3.3	2.2 ± 0.3	3.8 ± 2.1
	*NT min/max* (Δ)	−*2.5/−10.4* (*7.9*)	−*2.6/*−*5.4* (*2.8*)	−*2.9/*−*10.1* (*7.2*)	−*4.5/*−*8.3* (*3.8*)	−*3.3/*−*8.0* (*4.7*)	−*2.0/*−*4.6* (*2.6*)	−*2.2/*−*11.6* (*9.4*)

LT-values are means of 3–6 replicates. LT_50_ values and statistical indication are given in bold: different small letters in a line indicate statistical difference in frost resistance among the different species, different capital letters in a column indicate statistical difference in frost resistance among different development stages within a species; italics value shows significantly different statistical groups on the basis of *α* = 0.05 (one-way ANOVA, Bonferroni post hoc comparison, *α* = 0.05)

*ND* not determined

During the *b2* stage (bolting), frost resistance decreased significantly, and in most species temperatures around −2 °C already led to first frost damage (LT_10_) concomitant to ice formation in the tissues (Fig. 1). However, in *L. procumbens* and *R. glacialis*, *b2* buds were significantly more frost resistant than in the remainder of the species (LT_10_ −5.8 °C and −7.2 °C, respectively); (Table 3; Fig. 1). In *L. procumbens*, effective anatomical ice barriers prevent ice intrusion into the flower (G. Neuner et al., unpublished), and the reproductive shoot of *R. glacialis* is ice tolerant. Usually, frost-damaged *b2* buds became brownish and lost their turgor within a few days. In cushion plants, particularly in *S. bryoides,* buds often appeared intact after freezing for about 2 weeks and longer. Elongation of the flower stems continued; however, the stems became contorted and flower buds did not develop further.

During anthesis, depending on the species, frost resistance remained at approximately the same level as during bolting (*S. caesia*, *S. moschata*), increased (*R. ferrugineum*, *C. uniflorum*, *S. bryoides*), or decreased (*L. procumbens*, *R. glacialis*); (Table 3; Fig. 1). Among all species, inflorescences of *S. caesia* were the most frost susceptible (LT_50_ = −2.7 °C), those of *R. glacialis* the most frost resistant (LT_50_ = −8.0 °C). The flowers of most species did not tolerate ice formation in their tissues except for *R. glacialis* whose inflorescences remained ice tolerant during anthesis, and those of *R. ferrugineum* which seemed to endure at least some ice. During the fruiting phase, frost resistance increased again in most species. Except for *R. ferrugineum* and *S. bryoides,* reproductive shoots in the *f1* stage usually tolerated ice formation without frost damage (Fig. 1).

All in all, the *b2* stage and anthesis turned out to be the most frost susceptible, and *b1* and the fruiting stage the most frost-resistant stages during reproductive development (Fig. 2a). Pooling all stages of development together in different species groups, frost resistance (mean LT_50_) significantly differed with regard to flowering times (Fig. 2b), among growth forms (Fig. 2c), and between the alpine and nival species group (Fig. 2d): early flowering species (−7.5 °C) tolerated lower temperatures than mid (−5.1 °C) and late (−4.9 °C) flowering species (*p* < 0.000, one-way ANOVA); herbs (−6.9 °C) were less frost susceptible than woody plants (−5.4 °C) and cushion plants (−4.8 °C; *p* ≤ 0.010, one-way ANOVA); and nival species (−6.0 °C) were frost hardier than alpine species (−4.9 °C; *p* = 0.001, *t* test).

The temperature range between 10 and 90 % damage (ΔLT_10_–LT_90_) of reproductive shoots was highly variable (Table 3) and, depending on the species and on the reproductive stage, ranged from 2 to 9 K, being significantly narrower in species restricted to the alpine zone (2.9 K on average) than in nival species (3.9 K on average) (*p* = 0.047, *t* test). The temperature range wherein ice nucleation events were registered (*NT* Δ *min*–*max*; Table 3) was between about 3 K (*S. caesia*) and 8 K (*S. moschata*) indicating different capacities for supercooling (see also Fig. 1).

### Vegetative shoots versus reproductive shoots

In all species, except for *R. glacialis*, reproductive shoots were significantly more frost susceptible than vegetative shoots (Fig. 1). Averaged over all species and developmental stages, the difference was 4.2 K ± 2.7 SD. Taking all species together (see Fig. 2a), the difference tended to be more pronounced during the *b2* stage (4.9 K) and anthesis (4.4 K) than during the *b1* stage (3.7 K) and fruiting (3.6 K); however, the differences were not significant (*p* = 0.25; one-way ANOVA). Pooling all reproductive stages, the difference was smaller in early flowering species (1.7 K) than in mid- (3.5 K) and late flowering species (5.8 K); (*p* < 0.001, one-way ANOVA; Fig. 2b). Furthermore, the difference was smaller in herbs (2.4 K) and woody plants (3.3 K), than in cushion plants (5.8 K; *p* < 0.001, one-way ANOVA; Fig. 2c). However, the difference was the same (4.2 K) in the alpine and nival species group (Fig. 2d).

### Reproductive success

The lower the treatment temperature the more flower buds, flowers and young fruits suffered from frost damage and did not mature into fruits. Depending on the species and on the timing of the frost treatment, first fruit losses (i.e. the Fr/Fl ratio dropping below control values) resulted from exposure to temperatures between −2.2 and −7.0 °C, and full fruit loss occurred from −6.0 to −15.0 °C (Table 4). Fruits that had attained maturity within the temperature range causing first to almost full losses were further inspected for seed set. A correlation between the treatment temperature and the S/O ratio was either missing or very low (average Pearson *r* = 0.13 ± 0.08 SD). This means that seed set in fruits that had been frost-treated at an earlier stage of reproductive development and had remained undamaged, did not significantly differ from seed set in control fruits.

**Table 4:** Effect of exposure to freezing temperatures at different stages of reproductive development (*b1* before bolting; *b2* bolting; *a* anthesis; *f1* early fruit development) on the reproductive success of the investigated plant species

Stage of development	Dwarf shrubs		Herbs		Cushion plants		
	*Loi pro*	*Rho ferr*	*Cer uni*	*Ran gla*	*Sax bry*	*Sax cae*	*Sax mos*
*b1*	ND	−4.5/−6.8	ND	ND	−6.5/−12	−3.6/−11	−4.5/−14
*b2*	−5.2/−15	−4.5/−7.7	−2.8/−10.5	−7.0/−11.8	−2.2/−8.6	−2.3/−7.1	−2.7/−10.7
*a*	−5.0/−8.5	−4.7/−8.0	−4.4/−7.4	−6.0/−10.6	−3.3/−7.2	−2.2/−6.0	−3.2/−8.9
*f1*	−5.2/−10.2	−2.4/−6.4	−6.5/−10.1	−5.4/−9.7	−3.3/−10.6	−5.3/−6.0	−3.8/−11.0

Indicated are the temperatures (°C) at the first drop of the Fr/Fl ratio below the control value of untreated plants and at full fruit loss

*ND* not determined

Similar results hold true for seed germination in *S. caesia* and *C. uniflorum* after frost treatments during the *f2* stage. Germinability slightly decreased with decreasing temperature, both in laboratory tests and at the natural sites; however, there was only a weak correlation (*r* ≤ 0.3, Pearson).

### Site temperatures and the risk of frost damage

**Fig. 3:**
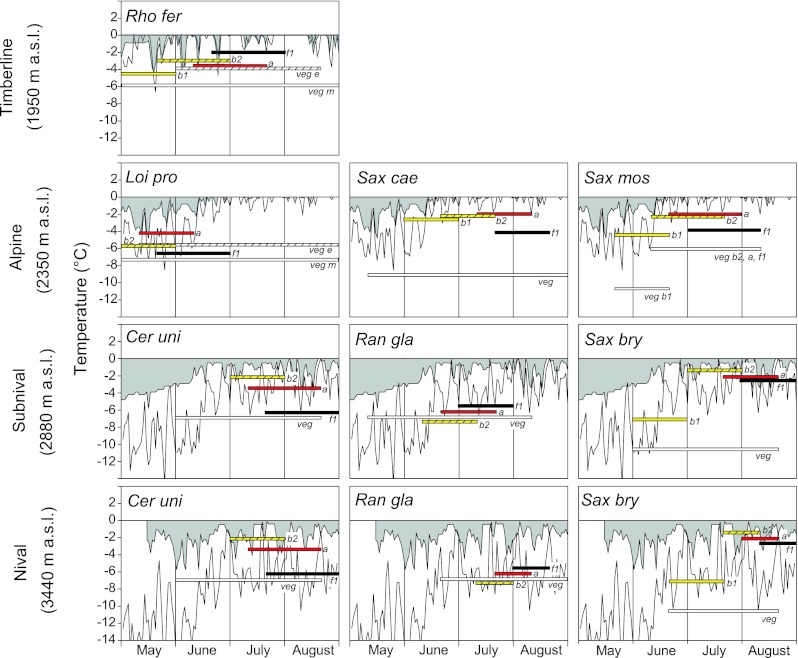
Absolute temperature minima recorded during multiannual periods in different habitats and frost resistance (LT_10_) in different stages of reproductive development.* Lower border of the grey area* absolute daily minima in the plant canopy;* thin line* absolute daily air temperature minima (2 m).* Coloured bars* show the duration of the main stages of development. Reproductive shoots: *yellow* bud stage *b1*; *yellow hatched* bud stage *b2*; *red* anthesis;* black* early fruiting *f1*. Vegetative shoots (*veg*) *white*; for the dwarf shrubs *Rho fer* and *Loi pro* *white* stands for mature shoots (*veg m*) and* white hatched* for expanding young shoots (*veg e*)

**Fig. 4:**
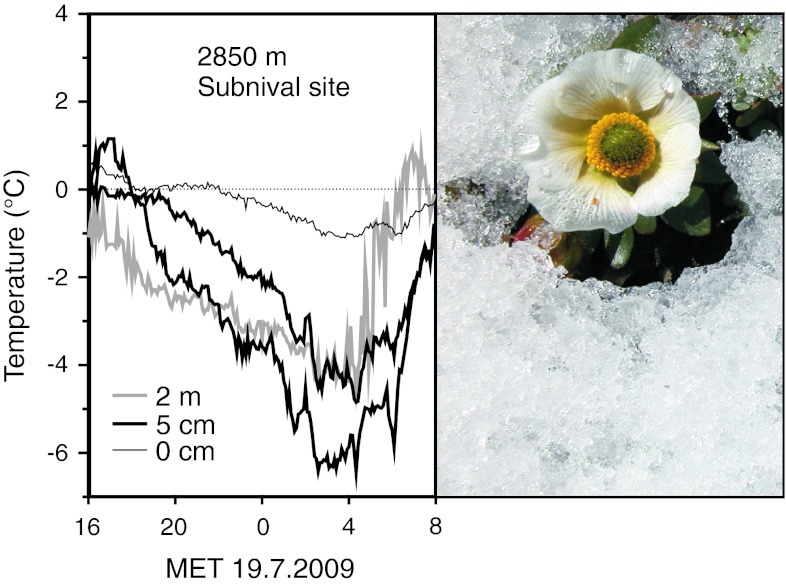
Diurnal course of temperatures after a cold spell in summer (19 July 2009) at the subnival site (Stubai Glacier, 2,880 m a.s.l.) recorded with thermocouples in the plant canopy near the ground (0 cm; *thin line*), 5 cm above the ground (*thick lines*) and 2 m in the air (*grey line*). Near the ground, plants are protected by snow and experience only slight frost, whereas plant parts that rise over the snow cool down to air temperatures or, because of radiative cooling, even lower. The photograph on the *right* illustrates the situation on the example of *R. glacialis* (the photo was taken after a cold spell on 13 July 2005 at the same site)

**Fig. 5:**
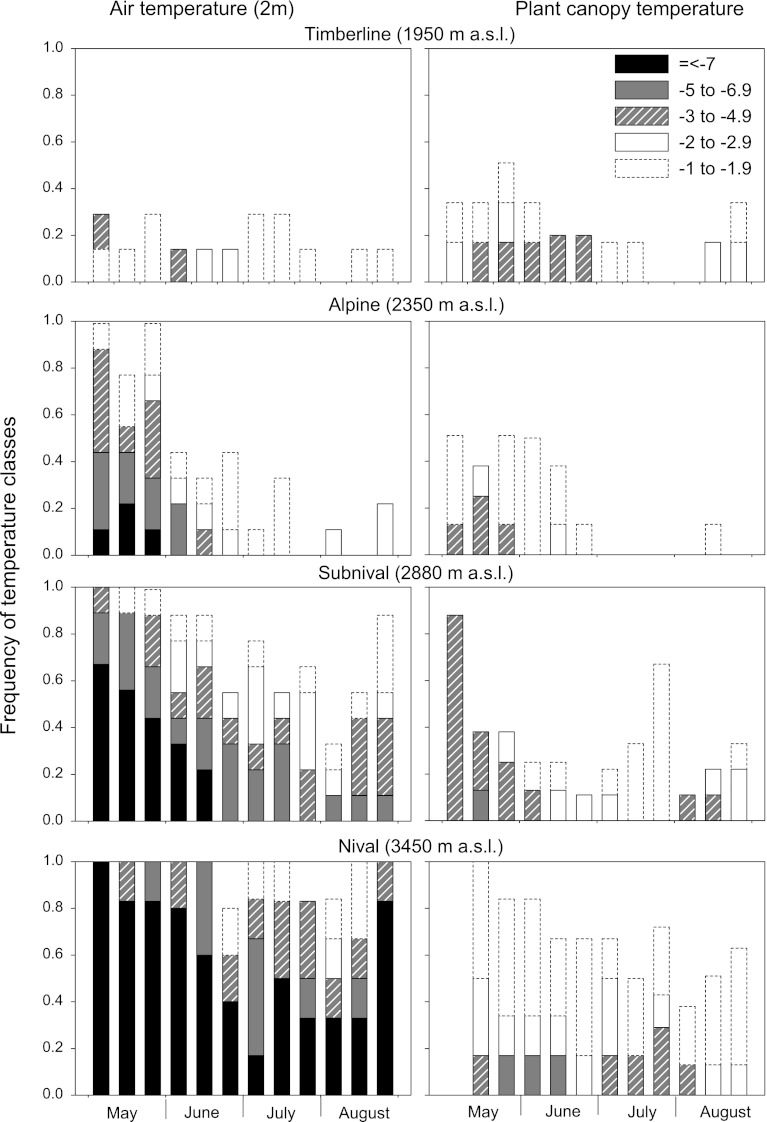
Frequency of temperature minima during the growing season in different mountain habitats. *Bars* show the proportion of different temperature classes below −1 °C for the first, middle and last third of each month (*black* ≤−7 °C; *grey* −5 to −6.9 °C; *hatched* −3 to −4.9 °C; *open bars*, *solid line* −2 to −2.9 °C; *open bar*, *dotted line* −1 to −1.9 °C; free space above a bar up to the frequency value of 1 indicates the proportion of temperatures higher than −1 °C). Calculations are based on multiannual temperature records (for details, see “Site temperatures”)

During cold spells in summer, plants without snow protection are at a much greater risk of suffering from frost damage than those covered by snow (see Fig. 3 for threshold values of LT_10_ for frost damage during different reproductive stages of the studied species and absolute daily temperature minima during multi-annual periods). The risk of damage can be even greater when unprotected plant parts cool below the air temperature due to night-time radiation (Fig. 4). The empirical probability (*EP*) of frost damage (Table 5), however, is mainly determined by the frequency of frost events (Fig. 5).

**Table 5:** Empirical probability (*EP*) of vegetative and reproductive shoots sustaining frost damage during different reproductive stages at different sites

Species	Stage	LT_10_	*EP* without snow protection												*EP* with snow protection											
			May			June			July			August			May			June			July			August		
		Mean (°C)	1	2	3	1	2	3	1	2	3	1	2	3	1	2	3	1	2	3	1	2	3	1	2	3
*Loi pro*																										
Alpine	*veg e*	−5.7	**0.3**	**0.4**	**0.3**	***0.2***	*0*	*0*	*0*	0	0				0	0	0	*0*	*0*	*0*	*0*	0	0			
	*veg m*	−7.3	**0.1**	**0.2**	**0.1**	0	0	0	0	0	0	0	0	0	0	0	0	0	0	0	0	0	0	0	0	0
	*b2*	−5.8	***0.4***	***0.4***	***0.3***	**0.2**									*0*	*0*	*0*	0								
	*a*	−4.2	**0.9**	***0.6***	***0.7***	***0.2***	**0.1**								0	*0*	***0.1***	*0*	0							
	*f1*	−6.5		**0.4**	*0*	***0.2***	*0*	*0*	0							0	*0*	*0*	*0*	*0*	0					
*Rho fer*																										
Timberline	*veg e*	−3.8		**0.3**	0	***0.1***	*0*	*0*	*0*	*0*						**0.2**	**0.3**	***0.2***	***0.2***	***0.2***	*0*	*0*				
	*veg m*	−5.9	0	**0.2**	0	0	0	0	0	0	0	0	0	0	0	0	0	0	0	0	0	0	0	0	0	0
	*b1*	−4.5	***0.3***	***0.2***	***0.1***	**0.2**	0								*0*	***0.2***	***0.3***	**0.2**	**0.2**							
	*b2*	−3.0		**0.1**	***0.1***	***0.2***	*0*	*0*	0							**0.2**	***0.3***	***0.2***	***0.2***	***0.2***	0					
	*a*	−3.7				**0.2**	*0*	*0*	*0*	*0*	0							**0.2**	***0.2***	***0.2***	*0*	*0*	0			
	*f1*	−2.0					**0.1**	***0.2***	*0*	*0*	*0*	0							**0.2**	***0.2***	*0*	***0.3***	*0*	0		
*Cer uni*																										
Subnival	*veg*	−6.9				***0.4***	***0.4***	***0.3***	***0.2***	***0.3***	*0*	***0.1***	***0.1***	**0.1**				*0*	***0.1***	*0*	*0*	*0*	*0*	*0*	*0*	0
	*b2*	−2.1						**0.6**	***0.7***	***0.6***	***0.6***	**0.2**								**0.1**	***0.1***	*0*	*0*	**0.1**		
	*a*	−3.4							**0.3**	***0.4***	***0.2***	***0.1***	***0.4***	**0.4**							0	*0*	*0*	***0.1***	***0.1***	0
	*f1*	−6.3								**0.3**	*0*	***0.1***	***0.1***	***0.1***								0	*0*	*0*	*0*	*0*
Nival	*veg*	−6.9						***0.4***	***0.6***	***0.5***	***0.3***	***0.3***	***0.3***	***0.3***						*0*	*0*	*0*	*0*	*0*	*0*	*0*
	*b2*	−2.1								**0.9**	***0.8***	***0.7***	***0.7***	**1**								**0.2**	***0.4***	***0.1***	***0.1***	**0.1**
	*a*	−3.4									**0.8**	***0.5***	***0.7***	***1***									**0.3**	***0.1***	*0*	*0*
	*f1*	−6.3										**0.3**	***0.5***	***0.8***										0	*0*	*0*
*Ran gla*																										
Subnival	*veg*	−6.8			**0.7**	***0.4***	***0.4***	***0.3***	***0.2***	***0.3***	*0*	***0.1***	0	0			0	*0*	*0*	*0*	*0*	*0*	*0*	*0*	0	0
	*b2*	−7.2			**0.4**	**0.3**	***0.2***	*0*	*0*	0							0	0	*0*	*0*	*0*	0				
	*a*	−6.2				**0.4**	**0.4**	***0.3***	***0.2***	***0.3***	**0.1**	**0.1**						0	0	*0*	*0*	*0*	0	0		
	*f1*	−5.6					**0.4**	**0.3**	***0.2***	***0.3***	*0*	**0.1**	**0.1**						0	0	*0*	*0*	*0*	0	0	
Nival	*veg*	−6.8						***0.4***	***0.6***	***0.5***	***0.3***	***0.3***	***0.3***	***0.3***						*0*	*0*	*0*	*0*	*0*	*0*	*0*
	*b2*	−7.2							**0.2**	***0.5***	***0.3***	**0.3**									0	*0*	*0*	0		
	*a*	−6.2								**0.5**	***0.5***	***0.3***	**0.5**									0	*0*	*0*	0	
	*f1*	−5.6									**0.5**	***0.3***	***0.5***	**0.8**									0	*0*	*0*	0
*Sax bry*																										
Subnival	*veg*	−10.6		**0.3**	**0.2**	***0.3***	*0*	*0*	*0*	*0*	*0*	*0*	*0*	0		0	0	*0*	*0*	*0*	*0*	*0*	*0*	*0*	*0*	0
	*b1*	−7.1		**0.6**	**0.4**	***0.3***	***0.2***	*0*	0	0						0	0	*0*	*0*	*0*	0	0				
	*b2*	−1.4					**0.9**	**0.6**	***0.8***	***0.6***	***0.7***	**0.3**	**0.4**						**0.4**	**0.1**	***0.3***	***0.3***	***0.7***	**0.1**	**0.3**	
	*a*	−2.1								**0.6**	***0.6***	***0.2***	***0.4***	**0.6**								0	*0*	***0.1***	***0.3***	**0.2**
	*f1*	−2.6									**0.6**	***0.2***	***0.4***	***0.6***									0	***0.1***	***0.3***	***0.2***
Nival	*veg*	−10.6		**0.8**	**0.8**	**0.6**	**0.6**	***0.4***	***0.2***	***0.5***	*0*	***0.2***	***0.2***	**0.7**		0	0	0	0	*0*	*0*	*0*	*0*	*0*	*0*	0
	*b1*	−7.1					**0.6**	***0.4***	***0.2***	***0.5***	**0.3**								0	*0*	*0*	*0*	0			
	*b2*	−1.4								**1**	***0.8***	***0.8***	*1*	**1**								**0.5**	***0.7***	***0.4***	***0.5***	**0.6**
	*a*	−2.1									**0.9**	***0.7***	***0.7***	**1**									**0.4**	***0.1***	***0.1***	**0.1**
	*f1*	−2.6										**0.7**	***0.7***	***1***										**0.1**	***0.1***	***0.1***
*Sax cae*																										
Alpine	*veg*	−9.1	0	*0*	*0*	*0*	*0*	*0*	*0*	*0*	*0*	*0*	*0*	*0*	0	*0*	*0*	*0*	*0*	*0*	*0*	*0*	*0*	*0*	*0*	*0*
	*b1*	−2.6	**0.9**	**0.6**	**0.8**	***0.3***	***0.2***	***0.1***	0						**0.1**	**0.4**	**0.1**	*0*	***0.1***	*0*	0					
	*b2*	−2.2				**0.3**	**0.2**	***0.1***	*0*	*0*	0							0	**0.1**	*0*	*0*	*0*	0			
	*a*	−2.0							0	*0*	*0*	***0.1***	0	**0.2**							0	*0*	*0*	*0*	0	0
	*f1*	−4.2								0	*0*	*0*	*0*	0								0	*0*	*0*	*0*	0
*Sax mos*																										
Alpine	*veg (b1)*	−10.7	0	0	*0*	*0*	*0*	0							0	0	*0*	*0*	*0*	0						
	*veg (b2,a,f1)*	−6.0			**0.2**	**0.2**	*0*	*0*	*0*	*0*	*0*	*0*	0	0				0	*0*	*0*	*0*	*0*	*0*	*0*	0	0
	*b1*	−4.3	**0.9**	**0.6**	***0.7***	***0.2***	***0.1***	0							**0.1**	**0.3**	***0.1***	*0*	*0*	0						
	*b2*	−2.2			**0.8**	**0.3**	***0.2***	***0.1***	*0*	*0*	0	**0.1**					**0.1**	0	***0.1***	*0*	*0*	*0*	0	0		
	*a*	−2.1					**0.2**	***0.1***	*0*	*0*	*0*	**0.1**	0						**0.1**	*0*	*0*	*0*	*0*	0	0	
	*f1*	−4.0						0	*0*	*0*	*0*	*0*	0	0						0	*0*	*0*	*0*	*0*	0	0

Calculations were made on the basis of the frequency of freezing air temperatures at 2 m (reflecting frost acting on plants exposed to free air) and in the plant canopy near the ground below the snow in the respective habitat. Numbers give the empirical probability of suffering from frost damage (based on LT_10_) for the first, middle and last third of each month in which the respective stages of development occur. Probability range: 0 (i.e. the probability of frost damage is zero) to 1.0 (i.e. the probability of frost damage is 100 %). An *EP* of, e.g., 0.4 means that statistically damage is to be expected in 4 years out of 10. Values for *EP* > 0 are printed in bold. Values (bold and non-bolded) in italics mark the main period for each reproductive stage and for vegetative shoot growth, respectively (according to Larl 2007; Ladinig and Wagner 2009; Wagner et al. 2010, 2012; and observations in the present study)

Reproductive stages: *b1* before bolting; *b2* bolting; *a* anthesis; *f1* early fruiting. Vegetative stages: *veg e* vegetative expanding young shoots, *veg m* vegetative mature shoots, in *S. moschata* vegetative shoots are differently susceptible to frost before bolting (*b1*) and during later reproductive stages (*b2,a,f1*)

*Rhododendron* shrubs at the timberline are mostly insufficiently protected by snow during summer cold spells and thus are exposed to air temperatures or, because of radiative cooling, even lower temperatures (cf. Fig. 5 timberline; air versus plant temperature). The empirical probability of frost damage is almost zero in mature vegetative shoots, but is 0.2–0.3 in young shoots during the flushing period—which means, statistically speaking, frost damage in 2–3 years out of 10 (Table 5). Similarly, this holds true for reproductive shoots. Young fruits are particularly frost susceptible (mean LT_10_ −2 °C) and the empirical probability of suffering frost damage is 0.3 even in July.

At the alpine site (2,350 m a.s.l.), vegetative short-stem shoots of the cushion plant *S. caesia* are at no time endangered by frost, while in *S. moschata* there is only a slight risk of frost damage in the event of an early start to the growing season (Table 5). Reproductive *b1* and *b2* stages without snow protection are at a particularly high risk of frost damage in May (*EP* 0.6–0.9), whereas below the snow the frost damage risk is markedly lower (*EP* 0.1–0.4). Flowering and fruiting, which proceed mainly during July and August, are unlikely to be endangered by frost injuries. The prostrate-growing dwarf shrub *L. procumbens* is safe when covered by snow but is highly endangered when unprotected. The empirical probability is 0.3–0.4 for early flushing vegetative shoots and 0.4–0.7 for reproductive shoots before and during anthesis.

At the subnival and nival sites (2,880 and 3,440 m a.s.l.), severe summer frosts occur more frequently (Fig. 5). Without snow protection, the probability that reproductive shoots are frost damaged during the main reproductive period is up to 100 % in *S.* *bryoides* and *C. uniflorum*, and still as high as 50 % in *R. glacialis* (Table 5). When covered by snow, *R.* *glacialis* is not endangered by frost events at any time and *C. uniflorum* only to a small extent (*EP* mostly 0–0.1 in the subnival zone, and up to 0.4 in the nival zone immediately before anthesis). *S. bryoides*, in contrast, still has a high risk of suffering frost damage particularly during bolting (LT_10_ −1.4 °C; *EP* up to 0.7).

## Discussion

### Frost resistance in different reproductive stages

Frost resistance of vegetative shoots—except for newly sprouting shoots in the woody species—did not change significantly during different reproductive stages. Depending on the plant species, freezing temperatures between −4 and −10 °C were survived without frost damage, which is in the line with earlier findings on plants from the European Alps (Larcher and Wagner 1976; Sakai and Larcher 1987; Körner 2003; Taschler and Neuner 2004; Taschler et al. 2004; Larcher et al. 2010). Reproductive shoots were not only clearly more frost susceptible than vegetative shoots but were also differently frost susceptible during different developmental phases. Reproductive buds before bolting and immature fruits tolerated extracellular freezing to a certain extent. During bolting and anthesis, however, ice tolerance was lost. Exceptions were, on the one hand, *R. glacialis* which remained more or less ice tolerant during all reproductive stages, and, on the other hand, *R. ferrugineum* and *S. bryoides*, which also remained highly frost susceptible during early fruiting. The weakest link within the reproductive structures determines whether or not reproductive development continues after a frost event (Zinn et al. 2010). Detailed investigations in a separate study (Neuner et al. 2013) have shown that the peduncle including flower stalks, the stigma and the style are the most frost susceptible reproductive structures. During expansion growth, cells clearly have little tolerance of extracellular freezing and the associated freeze-dehydration (Sakai and Larcher 1987; Taschler et al. 2004; Neuner and Beikircher 2010). Ice nucleation in the peduncle automatically led to full fruit loss in the affected inflorescence, which in the most frost susceptible species occurred from about −2 °C onwards. The critical temperature for a complete failure, however, was markedly lower in all species. Interestingly, for seed output and germination, the all-or-nothing principle seems to apply. As long as a flower or fruit remained undamaged, the seed/ovule ratio and the germination capacity did not significantly differ from control values.

### Frost resistance and altitude

Alpine and nival plant species differ in their altitudinal distribution centres (Braun-Blanquet 1954; Reisigl and Pitschmann 1958; Ozenda 1988; Pauli et al. 1999) and occupy different climatic niches (Gottfried et al. 2011). Nival plants are particularly snow tolerant, cope better with short growing seasons at generally lower temperatures and are better adapted to the increasing severity of frost than alpine plant species (Gottfried et al. 2002). Taschler and Neuner (2004) found a significant correlation between summer frost resistance of vegetative shoots and the upper distribution boundary of a species. We expected the same for reproductive shoots. However, only *b1*-buds showed a significantly higher frost resistance with increasing upper distribution boundary (*r* = −0.92 for LT_50_, *p* = 0.04; Pearson), which reflects adaptation to the strong frosts still occurring at the beginning of the growing season. During bolting, anthesis and early fruiting, no clear relationship between frost resistance and altitude exists. For example, the reproductive shoots of the investigated saxifrages are similarly frost susceptible, irrespective of whether the species is restricted to the alpine zone (*S. caesia*) or occurs up to the nival zone (*S. bryoides*, *S. moschata*). Obviously, evolutionary adaptation to frost is limited during stages of cell expansion, tissue specialization and the complex sequence of sexual functions. The weakness of an inadequate frost adaptation during the functional phase of the flower seems to be partly compensated for through phenological adjustment in that frost susceptible stages of reproductive development are placed in periods with reduced risk of frost damage (Totland 1997; Inouye 2000; Körner 2003; Kudo and Hirao 2006). This strategy also becomes evident in the species we investigated which, irrespective of their distribution centre, flower later the more ice sensitive are their reproductive structures (compare Table. 1; Fig. 1).

Overall, it appears that ice tolerance in reproductive structures is an advantage (see *R. glacialis*) but not an absolute precondition for colonising high altitudes with frequent frost events. High-mountain plants are mainly slow-growing perennials which can live for several decades or even centuries (Morris and Doak 1998). Thus, the individual lifetime reproductive success might be more important for the maintenance of a species than a regular seed output each year.

### Mechanisms of frost survival during summer cold spells

Temperature minima occurring during summer cold spells in mountains regularly exceed the threshold values for frost damage in reproductive shoots during sensitive developmental phases. But this does not necessarily result in total damage of reproductive structures and full seed loss. Depending on the plant species and on the developmental stage, there is a more or less broad temperature range between first and severe frost damage. For the cushion plants *Saxifraga bryoides*, *S. caesia*, *S. moschata* and *Silene acaulis*, Hacker et al. (2011) could show that ice nucleation occurs independently in each single reproductive shoot—mainly in the stalks, and less frequently in the flower buds and flowers—and ice does not propagate into neighbouring shoots. Anatomical ice barriers have not yet been detected, which suggests that the dense cushion structure provides a thermal block for ice propagation (Hacker and Neuner 2008). Independent freezing events limited to single reproductive shoots increase the chance of supercooling and thus the chance of survival for the remaining shoots (Hacker et al. 2011). Supercooling of up to 7.8 K was observed during bolting, and up to 6.5 K during anthesis in cushion plants tested here. A similarly wide temperature span of 8.4 K in *C. uniflorum* during the bud stage suggests separate freezing of single shoots which might be facilitated by its compact thermally insulating cushion-like structure. During anthesis and fruiting, when the growth habit of *C. uniflorum* becomes much looser, the temperature span is reduced to 2.8 K which might indicate rapid freezing throughout upon ice nucleation. In *L. procumbens*, anatomical ice barriers may allow supercooling of the inflorescences and thus protect flowers from ice intrusion (Neuner et al., unpublished). In contrast to the other species, reproductive shoots of *R. glacialis* are ice tolerant, and do not have ice barriers (Hacker et al., unpublished). Ice spreads throughout at temperatures as high as −3 °C; however, frost damage mostly occurs at distinctly lower freezing temperatures. Ice tolerance is a more secure mechanism to survive frost events than supercooling which strongly depends on the actual site conditions such as the temperature gradient between the plant, the soil and free air, and, further, wind, wetting of leaves and potential extrinsic ice nucleation (Aryal and Neuner 2010). Ice tolerance in vegetative and reproductive shoots may be one of the reasons why *R. glacialis* is so successful in colonising frost-dominated habitats.

### Threats arising from frost events at the natural sites

The risk of frost injury depends largely on whether or not during summer cold spells plants are covered by snow. This becomes clear when frost resistance is compared with actual temperature minima during the growing season in the respective habitat. Below the snow, vegetative shoots of small-statured plants are at low risk of suffering from damage by summer frosts even at higher altitudes. Similarly, this holds true for reproductive shoots of the study species growing in the alpine zone. In this habitat, the only critical period is at the beginning of the growing season in May when temperatures may still drop down to −8 °C and the thinning winter snow cover does not sufficiently protect the flower buds. Later in the season, frost damage becomes unlikely—at least for *L.* *procumbens*, *S. caesia* and *S. moschata*—as temperature minima do not drop below the frost damage threshold. The situation is different in the subnival and nival zone, where frost susceptible reproductive shoots may become frost damaged even when covered by snow. Without snow protection, absolute temperature minima would cause frost damage to all nival species tested.

However, absolute daily temperature minima do not say much about the actual injury risk in the respective mountain habitat. The crucial point is the potential frequency with which frost susceptible stages are struck by injurious freezing temperatures. Probability calculations (cf. Table 5) on the basis of the climate of the last decade have shown that, in the case of adequate snow protection, frost damage in summer is more the exception than the rule, even in the nival zone. Such an exception is *S.* *bryoides*, for which the empirical probability of frost damage below the snow is up to 0.7 (i.e. frost damage is to be expected in 7 years out of 10) during bolting, which is in line with our observations at the natural growing sites (Ladinig and Wagner 2007). Outside the snow, the empirical probability of frost damage occurring during one of the reproductive stages is around 0.3 for *R. ferrugineum* at the timberline. However, it is already up to 0.7 in the alpine zone for early flowering species such as *L. procumbens*, and up to 1 (i.e. frost damage each year) for species growing in the nival zone.

In the European Alps—as in other mountain systems all over the world—there is a trend towards earlier snowmelt (mainly due to a reduced winter snow cover) and thus an earlier beginning of the growing season (Beniston 1997; Scheifinger et al. 2003), which increases the number of frost events early in the season (Baptist et al. 2010). An earlier start to the growing season leads to an earlier frost dehardening and thus exposes plants in frost-susceptible reproductive stages to an increased risk of frost damage—as already reported for several species in the Rocky Mountains (Inouye and Wielgolaski 2003; Inouye 2008), in the New Zealand Alps (Bannister et al. 2005), in the sub-arctic (Molau 1996), and in the European Alps after experimentally manipulating winter snow cover duration (Wipf et al. 2009). Modelling on the basis of topographic descriptors, microclimate data and vegetation records predicts marked changes in distribution patterns for individual species, species groups and communities in the alpine-nival ecotone of the Alps (Gottfried et al. 1998). In the context of reproduction, the persistence of a species in a location will also depend on whether sufficient individuals occur in safe, i.e. snow-protected, sites to ensure recruitment by seeds.
